# Galactose epimerase deficiency: lessons from the GalNet registry

**DOI:** 10.1186/s13023-022-02494-4

**Published:** 2022-09-02

**Authors:** Britt Derks, Didem Demirbas, Rodrigo R. Arantes, Samantha Banford, Alberto B. Burlina, Analía Cabrera, Ana Chiesa, M. Luz Couce, Carlo Dionisi-Vici, Matthias Gautschi, Stephanie Grünewald, Eva Morava, Dorothea Möslinger, Sabine Scholl-Bürgi, Anastasia Skouma, Karolina M. Stepien, David J. Timson, Gerard T. Berry, M. Estela Rubio-Gozalbo

**Affiliations:** 1 Department of Pediatrics and Clinical Genetics, Maastricht University Medical Centre+, P. Debyelaan 25, P.O. Box 5800, 6229 HX Maastricht, The Netherlands; 2 GROW, Maastricht University, Maastricht, The Netherlands; 3 MetabERN: European Reference Network for Hereditary Metabolic Disorders, Udine, Italy; 4 UMD: United for Metabolic Diseases Member, Amsterdam, The Netherlands; 5 Division of Genetics and Genomics, Harvard Medical School, Boston Children’s Hospital, 3 Blackfan Circle, Center for Life Science Building, Suite 14070, Boston, MA 02115 USA; 6 Special Service of Medical Genetics, Hospital das Clínicas da Universidade Federal de Minas Gerais, Belo Horizonte, Brazil; 7 South Eastern Health and Social Care Trust, Downpatrick, BT30 6RL UK; 8 Division of Inherited Metabolic Diseases, University Hospital, Via Orus 2/B, 35128 Padua, Italy; 9 Nutrition Department, Hospital de Niños V.J. Vilela, Sante Fe, Rosario, Argentina; 10 Department of Endocrinology, Hospital de Niños Ricardo Gutièrrez, Buenos Aires, Argentina; 11 Metabolic Unit, IDIS, Department of Neonatology, University Clinical Hospital of Santiago de Compostela. Calle Choupana, s/n, 15706 Santiago de Compostela, Spain; 12 Division of Metabolism, Bambino Gesu Children’s Research Hospital IRCCS, Piazza S Onofrio 4, 00165 Roma, Italy; 13 Division of Paediatric Endocrinology and Metabolism, Department of Paediatrics, University Hospital Bern, Inselspital, Freiburgstrasse 15, CH-3010 Bern, Switzerland; 14 Metabolic Medicine Department, NIHR Biomedical Research Center (BRC), Institute for Child Health, Great Ormond Street Hospital, University College London, London, UK; 15 Department of Clinical Genomics and Department of Laboratory Medicine and Pathology, Mayo Clinic, Rochester, MN USA; 16 Department of Pediatrics and Adolescent Medicine, Medical University of Vienna, Waehringer Guertel 18-20, 1090 Vienna, Austria; 17 Clinic for Pediatrics I, Inherited Metabolic Disorders, Medical University of Innsbruck, Anichstrasse 35, 6020 Innsbruck, Austria; 18 Institute of Child Health, Aghia Sophia Children’s Hospital, Thivon & Papadiamantopoulou, 11527 Athens, Greece; 19 Adult Inherited Metabolic Disorders Department, Salford Royal NHS Foundation Trust, Stott Lane, Salford, M6 8HD Greater Manchester UK; 20 School of Pharmacy and Biomolecular Sciences, University of Brighton, Huxley Building, Lewes Road, Brighton, BN2 4GJ UK

**Keywords:** Galactose epimerase deficiency, Galactosemia type III, Galactosemias Network, Galactose-restricted diet

## Abstract

**Background:**

Galactose epimerase (GALE) deficiency is a rare hereditary disorder of galactose metabolism with only a few cases described in the literature. This study aims to present the data of patients with GALE deficiency from different countries included through the Galactosemia Network to further expand the existing knowledge and review the current diagnostic strategy, treatment and follow-up of this not well characterized entity.

**Methods:**

Observational study collecting medical data from December 2014 to April 2022 of 22 not previously reported patients from 14 centers in 9 countries. Patients were classified as generalized or non-generalized based on their genotype, enzyme activities in different tissues and/or clinical picture and professional judgment of the treating physician.

**Results:**

In total 6 patients were classified as generalized and 16 as non-generalized. In the generalized group, acute neonatal illness was reported in 3, cognitive and developmental delays were present in 5 and hearing problems were reported in 3. Four generalized patients were homozygous for the genetic variant NM_001008216.2:c.280G > A (p.Val94Met). In the non-generalized group, no clearly related symptoms were found. Ten novel genetic variants were reported in this study population.

**Conclusion:**

The phenotypic spectrum of GALE deficiency ranges from asymptomatic to severe. The generalized patients have a phenotype that is in line with the 9 described cases in the literature and prescribing dietary interventions is the cornerstone for treatment. In the non-generalized group, treatment advice is more difficult. To be able to offer proper counseling, in addition to red blood cell enzyme activity, genetic studies, transferrin glycoform analysis and enzymatic measurements in fibroblasts are recommended. Due to lack of facilities, additional enzymatic testing is not common practice in many centers nor a tailored long-term follow-up is performed.

**Supplementary Information:**

The online version contains supplementary material available at 10.1186/s13023-022-02494-4.

## Introduction

Galactosemia type III (OMIM #230,350), also known as galactose epimerase deficiency or UDP-galactose-4-epimerase deficiency (GALE; EC 5.1.3.2), is one of the hereditary galactosemias [1], a group of inherited disorders of galactose metabolism. The GALE enzyme is the third enzyme in the Leloir pathway, the predominant route of galactose metabolism. Human GALE functions as a homodimer [2] and catalyzes the conversion of uridine diphosphate galactose (UDP-gal) to uridine diphosphate glucose (UDP-glc) [3, 4] maintaining an equilibrium ratio of UDP-Gal to UDP-Glc of one to three [5]. GALE also catalyzes the interconversion of uridine diphosphate-*N*-acetyl-galactosamine (UDP-GalNAc) and uridine diphosphate-*N*-acetyl-glucosamine (UDP-GlcNAc) [6], all necessary for the glycosylation of proteins and lipids [7].

The clinical presentation of GALE deficiency is considered a continuum ranging from a benign peripheral form to an intermediate form to a severe generalized form, depending on the affected tissues and the degree of GALE impairment [8–10].The benign peripheral form of GALE deficiency has an estimated prevalence of 1:6,700–1:60,000 and the generalized form is considered ultra-rare [10]. The peripheral form was first reported by Gitzelmann [11, 12], describing patients in whom GALE impairment was restricted to circulating red and white blood cells in combination with normal or near-normal levels of GALE in fibroblasts, liver, phytohemagglutinin (PHA) stimulated leukocytes and Epstein Barr virus (EBV) transformed lymphoblasts. In general, patients with the peripheral form are asymptomatic and undergo a normal growth and development despite raised galactose-1-phosphate (Gal-1-P) in the erythrocytes [1, 10]. The intermediate form has been defined as a deficient GALE enzyme activity in red and white blood cells with less than 50% of normal enzyme levels (not profoundly decreased) in other non-peripheral cells [9, 10]. Patients with the intermediate form show a variable clinical phenotype ranging from asymptomatic to neonatal transient illness, resolved upon dietary galactose restriction. However, their long-term outcome is still unclear [9]. The generalized form of GALE deficiency appears to be an extremely rare disorder, with only nine patients (five females and four males) of four families reported in the literature so far [13, 14]. In patients with generalized GALE deficiency, the enzyme activity is profoundly decreased in all tissues tested [9]. The first case of generalized GALE deficiency was reported in 1981, describing a newborn that presented on day five with a severe clinical picture similar to classic galactosemia and with a lack of GALE activity in red blood cells and fibroblasts [15]. Dysmorphic features as well as other long-term complications apparent from birth have been reported in other patients with generalized GALE deficiency [13, 14]. These patients are from highly consanguineous families, making it questionable which symptoms are attributable to the GALE deficiency.

Various genetic variants have been identified and described in the *GALE* gene located on chromosome 1p36.11 [16]. The most severe defects in GALE protein were observed in NM_001008216.2:c.280G > A (p.Val94Met), NM_001008216.2:c.269G > A (p.Gly90Glu) and NM_001008216.2:c.548 T > C (p.Leu183Pro) genetic variants. Homozygosity of NM_001008216.2:c.280G > A (p.Val94Met) has been found in the majority of patients with the generalized phenotype [17, 18].

In 2012, the international network of galactosemias (GalNet, https://www.galactosemianetwork.org) created a web-based patient registry including galactosemia type I, II and III [19]. This study aims to present the data of patients with GALE deficiency from different countries included through the GalNet network to further expand the existing knowledge and review the current practice diagnostic strategy, treatment and follow-up of this not well characterized entity.

## Results

### Patients’ characteristics

In this study, 22 patients, who were previously unreported in the literature with a median age of 9.5 years (range 7 months—37 years) were included. The majority, 77.3% (17/22) of patients were detected by newborn screening (NBS). There were 40.9% females and 59.1% males. The patients originated from 9 countries and 14 different centers (Table 1). Four patients came from a consanguineous family. Seventy-three percent of the patients were Caucasian (see Additional file 1). In 3 patients, additional genetic testing (Whole Exome Sequencing (WES)) was performed and revealed no other genetic variants. In total, 6 patients were categorized as generalized and 16 as non-generalized. The non-generalized group likely comprises patients with peripheral and intermediate forms. Due to the young population age, the development of long-term complication in asymptomatic patients could not be ruled out.

**Table 1:** Participating countries and center

Country	Center	NBS	Number of patients
Argentina	Hospital de Niños Ricardo Gutièrrez, Rosario	Yes	2
Austria	Universitätsklink für Pädiatrie, Tirol Kliniken GmbH, Innsbruck	Yes	1
	Medizinische Universität Wien Vienna	Yes	1
Brazil	Hospital das Clínicas da Universidade Federal de Minas Gerais	No	1
Greece	Institute of Child Health, Athens	Yes	3
Italy	Bambino Gesu Children’s Research Hospital, Roma	Yes	1
	Division of Inherited Metabolic Diseases, University Hospital, Padova	Yes	2
Spain	University Clinical Hospital of Santiago de Compostela	Yes	2
Switzerland	Insel spital, University Hospital, Bern	Yes	2
	University Children’s Hospital, Zürich	Yes	1
United Kingdom	Salford Royal NHS Foundation Trust Salford	No	3
	Great Ormond Street Hospital, London	No	1
USA	Boston Children’s Hospital	Yes	1
	Mayo Clinic, Rochester, Minnesota	Yes	1
Total			22

### Diet

All generalized patients followed a galactose-restricted diet initiated within the first month of life. Four of them followed a strict diet (lactose free and restrictions of non-dairy galactose). In the non-generalized group, 8 patients followed a galactose-restricted diet with onset within the first month of life in 7. Two non-generalized patients started a diet in the neonatal period, but diet was withdrawn during infancy. Six non-generalized patients did not follow a diet.

### Phenotypic spectrum

#### Neonatal illness

Acute neonatal illness was defined as having one of the following symptoms: icterus, encephalopathy (decreased consciousness with or without neurological symptoms), bleeding diathesis (abnormal prothrombin time (PT) and/or activated partial thromboplastin time (APTT)), infection signs or hypoglycemia (glucose < 2.6 mmol/L).

In the generalized group, 3 showed acute neonatal illness (see Additional file 1). These patients were not detected by NBS. In the peripheral group, acute neonatal illness was reported in 7 of the 16 patients, mainly due to the presence of icterus and/or hypoglycemia (see Additional file 1).

#### Long-term follow-up

Regarding the brain follow-up, developmental delay was reported in 5 of the 6 patients categorized as generalized, 4 suffered from both motor and mental delays and 1 suffered from motor delays. Language delay was reported in 4, speech disorders in 2 and learning disabilities in 2. Due to the lack of NBS in the corresponding countries, none of them were diagnosed following NBS. In these patients, GALE deficiency was suspected based on their clinical picture and after exclusion of classic galactosemia. Other reported neurological symptoms mentioned in the generalized group included general motor abnormalities in 2 and gait problems in 2 (see Additional file 1). In the non-generalized group, 1 patient suffered from gait problems (see Additional file 1).

Female gonadal follow-up was reported in 2 patients at the age of 24 and 34 years with generalized GALE deficiency. Neither of these patients showed delayed puberty or signs of primary ovarian insufficiency (POI) (see Additional file 1). Their menstrual cycles were regular and normal. Gonadal ultrasound revealed no abnormalities (see Additional file 1). Both patients have not yet tried to conceive. In the non-generalized group, information on the female gonadal follow-up was not available mainly due to the young population age.

Regarding the bone health, 2 of the 3 reported patients with generalized GALE deficiency showed decreased levels of vitamin D. In these 3 patients, a dual-energy x-ray absorptiometry (DEXA) was performed, which showed the presence of osteopenia (T-scores − 1.8) and of a lower bone density compared to peers (Z-scores: − 0.23, − 0.9, − 1.8). The physical activity was rated below World Health Organization (WHO) standards in these 3 patients (see Additional file 1). No bone fractures were reported. In total, 4 generalized patients used calcium and vitamin D supplements.

In the non-generalized group, vitamin D levels were measured in 5 patients, 3 of them showed vitamin D deficiency and all 3 did not follow a diet. No data of DEXA-scans was available. No bone fractures were reported. The physical activity was assessed in 7 patients, all within the normal levels according to the WHO standards. Eleven patients used vitamin D supplements, 1 used calcium supplements and 1 used both (see Additional file 1).

In addition, the presence of hearing impairments, hematological abnormalities and short stature were assessed. Hearing impairments were present in 3 generalized patients and 1 non-generalized patient. Hematological abnormalities were not reported in the generalized group. In the non-generalized group, one patient was reported with thrombocytopenia worsening with intercurrent infections. Short stature was present in 4 generalized patients and 1 non-generalized patient (see Additional file 1). In one generalized patient, low levels of IgM and IgA were found, regarded as of no clinical relevance.

### Metabolites

Data on metabolites is presented in Table 2. In the generalized group, data on neonatal Gal-1-P was present in 2 patients and was elevated in both. In 1 patient, the urinary galactitol was recently measured and was within the normal range. In 4 patients, glycosylation patterns of transferrin were analyzed to investigate the presence of glycosylation defects. One patient avoided dairy products from the start of birth and has never been on a strict diet. In the neonatal period, his transferrin revealed an abnormal pattern (type I congenital disorder of glycosylation (CDG)-pattern), which normalized after the neonatal period without dietary changes. Two other generalized patients showed abnormal type I CDG patterns before initiation of diet, which normalized after the diet was initiated. Surprisingly, one generalized patient showed normal transferrin patterns after the galactose-restricted diet was initiated a few hours in advance of the test sampling. In the non-generalized group, data on total galactose in blood was available in 11 patients, 7 showed elevated levels in the neonatal period/before diet. Neonatal Gal-1-P was measured in 11 patients and elevated in 8. In 6 patients the urinary galactitol was measured and was (near)-normal. Information on the transferrin patterns was available in 4 patients, which showed normal patterns before the initiation of a galactose restriction diet or without a diet.

**Table 2:** Metabolites in generalized and non-generalized patients

Patient	Genotype and enzyme activity	Total galactose in blood	Neonatal Gal-1-P	Urinary galactitol	Transferrin
		< 20 mg/dL	< 10 mg/dL or < 0.05 µmol/g Hb	2–81 mmol/mol creatinine	Pattern: normal/abnormal
*Generalized*					
P1	c.[280G > A];[284G > A] RBC: 8.3%	NR	NR	Recent – 11.0	Neonatal period – Abnormal type I pattern
					Most recent – normal
P2	c.[280G > A];[280G > A] RBC: 4.7%	NR	26 mg/dL	NR	Before initiation of diet – abnormal type I pattern
					After initiation of diet – normal
P4	c.[280G > A];[280G > A] RBC and fibroblast: undetectable	NR	44 mg/dL	NR	Before initiation of diet – abnormal type I pattern
					After initiation of diet – normal
P6	c.[632A > G];[820G > C] RBC and fibroblast: undetectable	NR	NR	NR	Few hours after diet initiation – normal
					After initiation of diet – normal
*Non-generalized*					
P7	c.[647C > T];[728A > C] RBC: 17.5%; lymphoblast: 40.1%	Neonatal – 43.0	3.4 mg/dL	Neonatal – 19.1	NR
				recent – 83.0	
P8	c. [755 T > C];[755 T > C] RBC: 4.1%	Neonatal – 14	2.9 µmol/grHb	NR	NR
		Recent – 11.8			
P9	c.[284G > A];[284G > A] RBC: 3.2%	Neonatal – 12.8	2.1 µmol/grHb	NR	NR
		Recent – 12.0			
P10	c.[449C > T];[449C > T] RBC: 0.0%	Neonatal – 62.9 recent – 6.4	10.8 mg/dL	NR	NR
P11	c.[646G > A];[646G > A]	Neonatal – > 50	NR	NR	NR
		Recent – 3.6			
P12	c.[796A > C];[538G > A]	Neonatal – 36.4	NR	NR	NR
		Recent – 1.6			
P13	c.[755 T > C];[290C > T]	Neonatal – 35.4	NR	NR	NR
		Recent – 2.1			
P14	c.[484 T > A];[820G > C] RBC: 33.3%	Before diet – 31	33.1 mg/dL	NR	NR
P15	c.[755 T > C];[755 T > C] RBC: 23.1%	Before diet – 19	27.9 mg/dL	NR	NR
P16	c.[318_319del];[658C > T]	Neonatal – 80	9.5 mg/dL	NR	NR
		Recent – 3.5			
P17	c.[647C > T];[602C > T] RBC: 6.4%	NR	51 mg/dL	Varies between 2 and 19	No diet – normal
P18	c.[647C > T];[602C > T] RBC: 4.5%	NR	69.7 mg/dL	Varies between 4 and 60	No diet – normal
P20	RBC 30.0%	NR	5.0 mg/dL	Recent – 1.17	NR
P21	c.[602C > T];[214G > A] RBC: 0.0%	NR	NR	Recent – 1	No diet – normal
P22	Heterozygous c.237G > ARBC: 1.7%; fibroblast: 31.4%	Recent –2.3 mg/dL	36.9 mg/dL	Neonatal – 10.0	Before initiation of diet – normal

*P* = patient; *NR* = not reported

### Enzyme measurement and genotypic spectrum

**Fig. 1:**
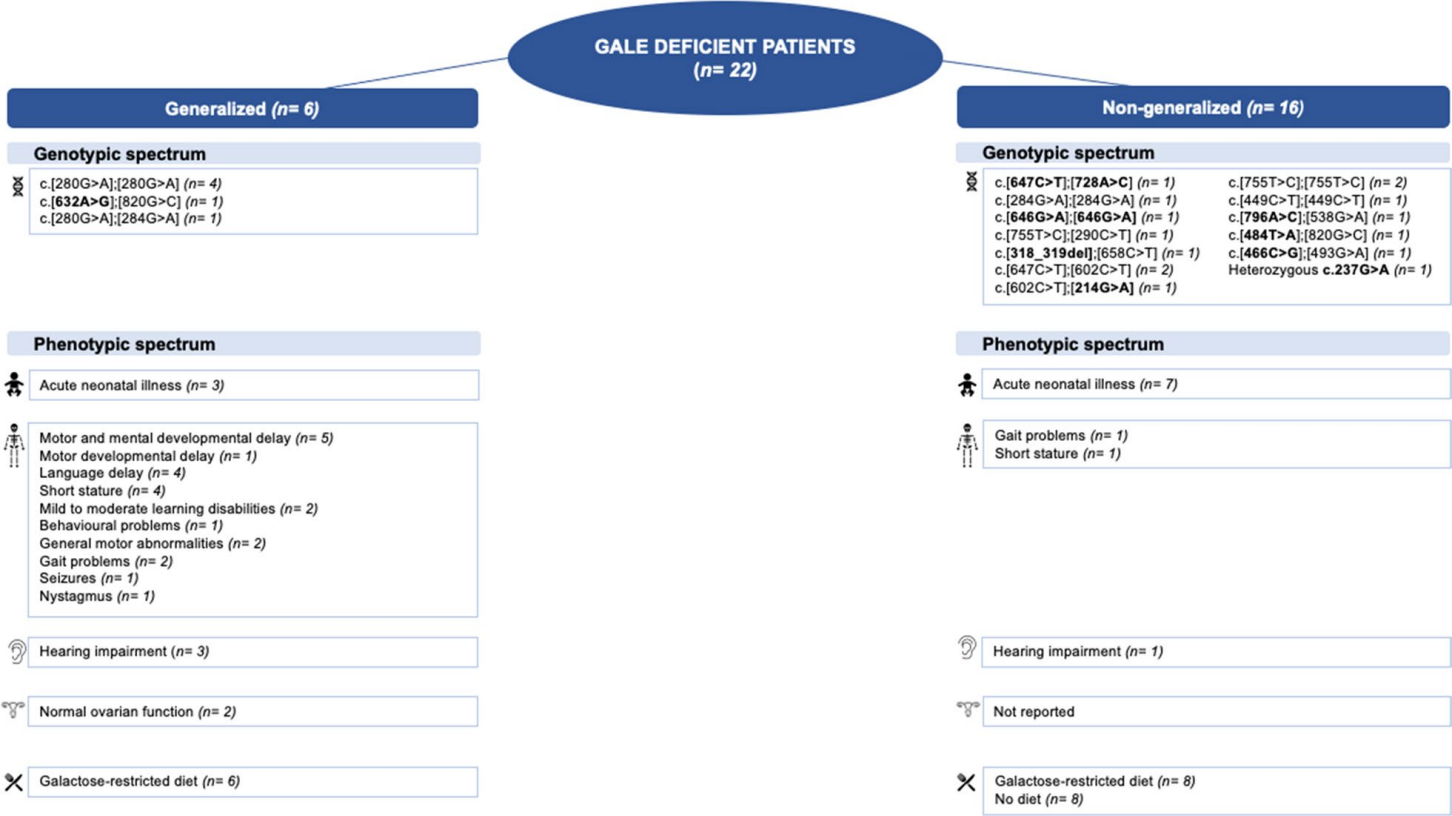
**Genotypic and phenotypic spectrum of the reported patients**. Patients were categorized into generalized or non-generalized. The number of patients per category is presented. The new genetic variants are showed in bold.  genotype;  neonatal period;  long term complications;  hearing problems,  ovarian function,  diet

The *GALE* gene variants (NM_001008216.2) were reported in 6 generalized patients and in 15 non-generalized patients. In the generalized group, 4 patients were homozygous for the variant NM_001008216.2:c.280G > A (p.Val94Met). Their enzyme activities measured in erythrocytes ranged from undetectable to 4.7%. In 2 patients, additional enzyme activities measured in fibroblasts were performed and were undetectable. One generalized patient was compound heterozygous for the variant NM_001008216.2:c.280G > A (p.Val94Met) and NM_001008216.2:c.284G > A (p.Gly95Asp) and showed an enzyme activity of 8.3% measured in the erythrocytes. The other generalized GALE deficient patient was compound heterozygous for NM_001008216.2:c.632A > G (p.Tyr211Cys) and NM_001008216.2:c.820G > C (p.Gly274Arg). In this patient, the GALE enzymatic level was undetectable in erythrocytes and fibroblasts (Fig. 1).

**Fig. 2:**
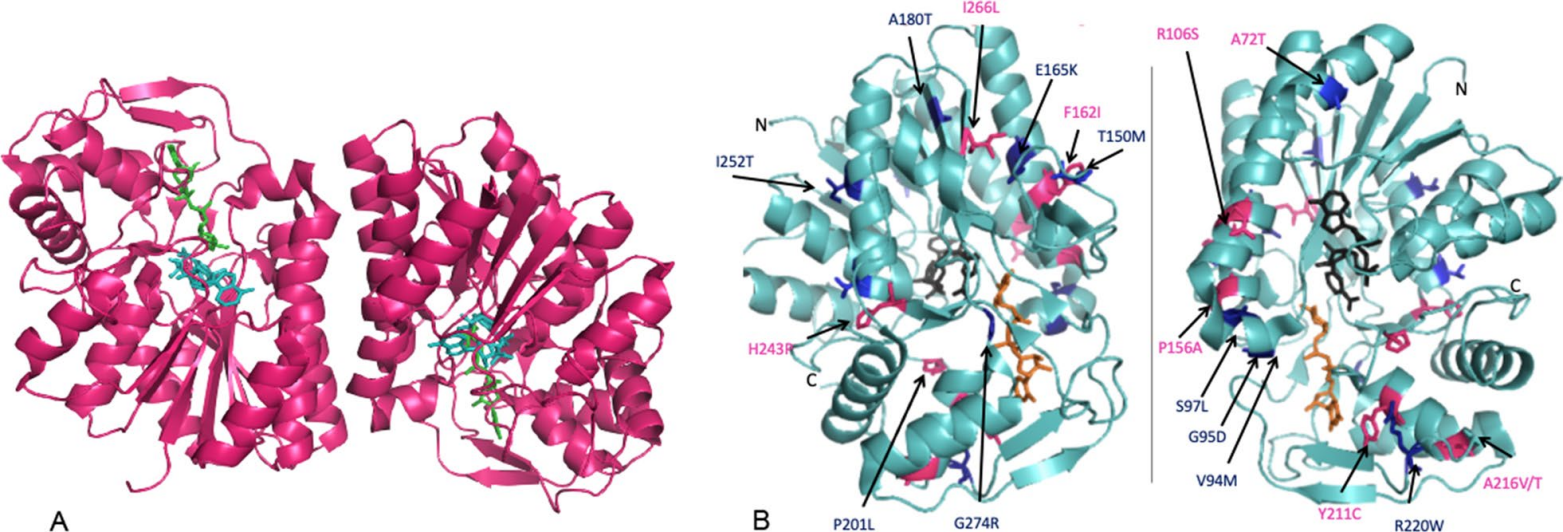
**Cartoon representations of the crystal structure of human GALE in complex with UDP-glucose and NADH**. **A** Crystallography of GALE enzyme in dimeric form **B** Two views of the monomeric protein with locations of genetic variants found in study population. Arrows depict the locations of the amino acids found to be altered in response to genetic variants seen in disease. Those shown in dark blue are missense genetic variants while those in pink are those unknown to gnomAD (Genome Aggregation Database). Figures were created in Pymol (www.pymol.org). PDB entry 1EK6 was used. *Thoden JB, Wohlers TM, Fridovich-Keil JL, Holden HM (2000) Crystallographic evidence for Tyr 157 functioning as the active site base in human UDP-galactose 4-epimerase. Biochemistry 39: 5691–5701*

In total, 10 unpublished genetic variants were reported in this study population, namely NM_001008216.2:c.466C > G (p.Pro156Ala), NM_001008216.2:c.632A > G (p.Tyr211Cys), NM_001008216.2:c.646G > A (p.Ala216Thr), NM_001008216.2:c.796A > C (p.Ile266Leu), NM_001008216.2:c.484 T > A (p.Phe162Ile), NM_001008216.2:c.318_319del (p.Arg106SerfsTer2), NM_001008216.2:c.647C > T (p.Ala216Val), NM_001008216.2:c.728A > C (p.His243Pro), NM_001127621.2:c.214G > A (p.Ala72Thr) and NM_001008216.2:c.237G > A (p.Lys79 =). The latter is a silent genetic variant affecting exon 4 and could therefore not be depicted in Fig. 2. A second variant in the *GALE* gene has not yet been identified in the patient with the silent genetic variant, but there may be another variant in the intronic regions. None of these unpublished variants were described on the Genome Aggregration Database (gnomAD; https://gnomad.broadinstitute.org/). In Fig. 2, these variants are depicted in the crystal structure of GALE in complex with UDP-glucose and NADH.

## Discussion

In this study, we describe the phenotypic and genotypic spectrum of 22 GALE deficient patients, 6 were classified as generalized and 16 as non-generalized.

### Phenotypic spectrum

So far, 9 individuals from 5 families are described in the literature with generalized GALE deficiency [13–15, 17, 20–22]. Patients with generalized GALE deficiency do develop acute clinical symptoms similar to classic galactosemia when they are exposed to galactose [9], which resolve when the patient is initiated on a galactose-restricted diet. However, despite the galactose-restricted diet, some patients with generalized GALE deficiency are reported with long-term complications. The majority of these reported patients showed hepatic abnormalities (8/9), short stature (7/7), developmental delay (6/6), hypotonia (6/8), sensorineural hearing loss (4/7), micrognathia (4/6), flexion deformities of the fingers (3/6), hip dysplasia (3/7), cataracts (3/8) and renal dysfunction (1/6) [9]. In our study population, we included 6 patients with generalized GALE deficiency from 4 different families. In Additional file 2, the clinical picture of these 6 patients compared to the 9 previously published patients with generalized GALE deficiency is summarized. The phenotype of our patients is comparable to the phenotype described in the literature. In our study population, the gonadal follow-up was reported in two female patients, sisters (age of follow-up 24 and 34 years) with generalized GALE deficiency. Interestingly, these patients did not show any signs of POI. This is in contrast to classic galactosemia, where 80% of the female patients suffer from POI [23], but is in line with previous findings of female patients with generalized GALE deficiency. Walter et al. (1999) [13] reported a female patient with generalized GALE deficiency that was severely affected, but did not show any signs of ovarian dysfunction. However, due to the small study population, these results should be interpreted with caution. Further research is needed to investigate whether or not there is a link between POI and GALE deficiency. Another resemblance between our study population and the described patients in the literature, is the high consanguinity rate, raising the question to what extent *GALE* variants or homozygosity for other autosomal recessive alleles were responsible for their phenotype and dysmorphic features. However, in our study population, a WES was performed in 3 generalized patients which revealed no other genetic variants than *GALE* variants.

Infants with intermediate GALE deficiency are usually asymptomatic in the neonatal period, even when they do not follow a galactose-restricted diet. However, in these patients the long-term outcomes and the effects of dietary interventions remain unclear [9]. A prospective follow-up could be helpful to answer the question whether dietary intervention is necessary. Alano et al. (1998) [24] described a GALE deficient patient following no specific diet that remained clinically well in the newborn period. At the age of 2 years, this patient developed intellectual and motor delays. The cause of the developmental delay was unknown, and it was stated that the influence of GALE deficiency could not be ruled out. However, in the general population, 15% of 3–17 years old children have at least one developmental delay [25].

Usually, newborns with peripheral GALE deficiency ingesting dairy milk are asymptomatic and are only detected if elevated levels of galactose are measured with NBS [9, 11]. Even without a diet, these patients appear to remain asymptomatic [9]. In our study population, 6 non-generalized patients were reported with acute neonatal illness, mostly based on the presence of icterus and/or or hypoglycemia. However, hypoglycemia and jaundice are also frequent in newborns in the general population [26, 27]. In our study population, the majority of non-generalized patients were asymptomatic and no clearly related symptoms to the GALE deficiency were found. However, due to the young age of the study population, it is difficult to exclude the development of symptoms on the long-term.

### Genotype

Due to the rarity of GALE deficiency, little is known about specific genotype–phenotype correlations. However, a few *GALE* genetic variants are clearly associated with a mild or severe phenotype. Homozygosity for the variant NM_001008216.2:c.280G > A (p.Val94Met) is associated with a severe phenotype [14]. This is in line with our findings, as 4 of the 6 patients with generalized GALE deficiency were homozygous for this variant. Timson et al. (2013) [28] found that this variant does not lead to changes in the dynamics and stability of the enzyme but does lead to change in the active site dynamics. Because of this change, the binding of the substrate and probably the cofactor could be less stable. Other genetic variants have also been described in the literature that are associated with the peripheral or intermediate form [10, 29]. In our study population, 9 previously unpublished variants were reported. It is difficult to predict the in vivo effect of genetic variants on the protein structure and function without molecular dynamic simulations or in vitro studies (see Fig. 2 for locations of residues affected by point mutations). Despite this, it is likely that changes in the residues that form part of the active site will have a more predictable effect on the protein function. The genetic variants NM_001008216.2:c.280G > A (p.Val94Met), NM_001008216.2:c.632A > G (p.Tyr211Cys) and NM_001008216.2:c.284G > A (p.Gly95Asp) all form part of the substrate binding site and are therefore likely to impact substrate binding. The substitution of a negatively charged Asp residue for the neutral Gly-95 will likely have a substantial impact. The NM_001008216.2:c.820G > C (p.Gly274Arg) and NM_001008216.2:c.493G > A (Glu165Lys) substitutions also involve a change in charge. Timson et al. (2013) [30] predicted that NM_001008216.2:c.493G > A (Glu165Lys) causes a severe variant due to its interaction with the Lys-161 in the active site. Change in the polarity of the parts of the protein chain (NM_001008216.2:c.755 T > C (p.Ile252Thr) and NM_001008216.2:c.290C > T (Ser97Leu)) could adversely affect protein folding. Changes in the residues of the dimer interface, as in NM_001008216.2:c.484 T > A (p.Phe162Ile) could cause disruption in the proteins’ ability to dimerize. Substitution of proline residues, as in NM_001008216.2:c.466C > G (p.Pro156Ala) and NM_001008216.2:c.728A > C (p.His243Pro) can be particularly harmful as proline normally ends an α-helix. The substitution of smaller residues for larger ones (NM_001008216.2:c.658C > T (p.Arg220Trp); NM_001008216.2:c.449C > T (p.Thr150Met)) can impact the flexibility of the protein. Proteins require optimal flexibility for full activity. NM_001008216.2:c.449C > T (p.Thr150Met) is thought to have an intermediate effect through its interaction with Ser-132 [30]. It is difficult to predict the structure changes in NM_001008216.2:c.646G > A (p.Ala216Thr), NM_001008216.2:c.647C > T (p.Ala216Val), NM_001008216.2:c.796A > C (p.Ile266Leu) and NM_001008216.2:c.538G > A (p.Ala180Thr) due to the relatively conservative nature of the variation.

Although the NM_001008216.2(GALE):c.237G > A variation generates a synonymous coding effect at the protein level (p.(Lys79 =); the variant itself is predicted to alter a splice donor site. It causes a decrease in splicing signal predicted by different algorithms (MaxEnt: − 51.5%, NNSPLICE: − 64.1%, SSF: − 14.2% and HSF: − 11.2%). The allele frequency for this variant is 0.018475% in the African/African American population and it is not observed in other populations (gnomAD v2.1.1).

Summarizing, the novel genetic variant NM_001008216.2:c.632A > G (p.Tyr211Cys) is probably associated with generalized GALE deficiency and the novel genetic variants NM_001008216.2:c.290C > T (Ser97Leu), NM_001008216.2:c.658C > T (p.Arg220Trp), NM_001008216.2:c.466C > G (p.Pro156Ala), NM_001008216.2:c.484 T > A (p.Phe162Ile) and NM_001008216.2:c.728A > C (p.His243Pro) are probably associated with the non-generalized (intermediate or peripheral) form of GALE deficiency.

### Diagnostic burden

When a GALE deficiency is suspected, the diagnosis can be established by diminished GALE enzyme activity in red blood cells (RBC) and/or by the identification of *GALE* pathogenic variants [9]. However, in an effort to classify the patient, additional GALE enzyme activities should be measured in fibroblasts or lymphoblasts. Enzymatic stability and catalytic efficiency of the GALE enzyme could be causative factors in the continuum of GALE deficiency [31], since patients with peripheral GALE deficiency show normal enzyme activity in liver and fibroblasts versus patients with generalized GALE deficiency who show profoundly decreased enzyme activity in other cell types, such as liver and fibroblasts [9, 32].

It is not usual practice in many centers to perform additional investigations in other tissues and or genetic testing to better classify the deficiency and consequently tailor the follow-up. GALE activity measurement such as in fibroblasts, genetic testing - preferably a WES when consanguinity is present – and metabolite testing is only available in a few centers.

Additional studies are desired in order to decide whether or not to initiate a galactose-restricted diet. In our study population, 8 patients classified as non-generalized – based on their clinical picture – do follow a galactose-restricted diet.

### Glycosylation studies

Glycosylation studies such as serum transferrin glycoform analysis, may be a valuable tool in determining whether dietary restrictions are necessary. In addition to its function in the Leloir pathway, GALE also catalyzes the interconversion of UDP-*N*-acetyl-galactosamine (UDP-GalNAc) and UDP-*N*-acetyl-glucosamine (UDP-GlcNac). Abnormal production of UDP-Glc, UDP-Gal, UDP-GalNAc and UDP-GlcNac can alter glycans [33, 34]. In generalized GALE patients, abnormal serum transferrin glycosylation patterns normalizing after the initiation of diet have been observed [13, 14]. The abnormal patterns found in GALE deficient patients are consistent with the serum transferrin glycosylation patterns in classic galactosemia. Sturiale, et al. (2005) [35] demonstrated partial deficiency of whole glycans of serum transferrin in classic galactosemia patients characterized by increased fucosylation and branching similar to congenital defects of glycosylation type I. These abnormalities of transferrin *N*-glycan biosynthesis restore after the initiation of diet [35].

Glycosylation is also important for the biogenesis of platelets and the homing of hematopoietic cells, glycosylation defects may be the cause for hematological abnormalities seen in a few GALE deficient patients. *N*-acetyllactosamine, a dimer of galactose and UDP-galNAc, is abundantly present on ß1-integrin, an important membrane protein on platelets for homing and extracellular interactions. Thus, GALE deficiency may lead to abnormal glycosylation of ß1-integrin causing either insufficient homing of megakaryocytes and platelet progenitor cells and impaired interaction with extracellular matrix. Seo et al. (2019) [36] reported 6 consanguineous related individuals with homozygosity for NM_001008216.2:c.151C > T (p.Arg51Trp) and severe thrombocytopenia. In addition to NM_001008216.2:c.151C > T (p.Arg51Trp), the variant NM_001008216.2:c.449C > T (p.Thr150Met) has also been associated with hematologic and immune abnormalities [37]. In our study, one patient with homozygosity for NM_001008216.2:c.449C > T (p.Thr150Met) was included and was reported with thrombocytopenia, which worsened during intercurrent infections. Thus, GALE should be suspected in patients suffering with thrombocytopenia, dysmegakaryopoiesis and hemolytic anemia [37].

### Newborn screening

In some countries, NBS includes GALE deficiency as part of screening for galactosemia, either as a secondary target disorder, or as an additional finding (see Table 1). This is possible if total galactose is measured as first tier parameter. GALE deficiency is suspected when newborn screening shows increased total galactose (specifically Gal-1-P), but normal GALT activity. Efforts to reduce the number of false positives of the screening for classic galactosemia tend to use GALT activity as exclusive first tier, which precludes screening for GALE deficiency [38]. From 1968 to 2019, 30 cases with GALE deficiency were found with increased total galactose and normal GALT activity in Switzerland. This equates to an estimated incidence of 1:133,604 (personal communication, Prof Matthias Baumgartner, medical head of the Swiss NBS program). Since the emergence of NBS for GALE deficiency in several countries, more patients are diagnosed with GALE deficiency. For an efficient and safe NBS, it will be important to be able to clearly distinguish between cases of purely peripheral GALE deficiency, which can be considered as a biochemical variant that does not need treatment and is thus in regard to NBS a false positive, and the generalized form that needs a galactose-restricted diet in order to prevent disease symptoms. However, for the intermediate form of GALE deficiency this is not yet clear.

### Metabolites

Patients with GALE deficiency are unable to synthetize UDP-gal by the pyrophosphorylase pathway and are therefore dependent on exogenous dietary galactose [14, 15]. On the other hand, dietary restriction of galactose is desired to prevent the development of acute symptoms. In the neonatal period, infants with GALE deficiency ingesting dairy milk show elevated Gal-1-P levels in the erythrocytes and elevated urinary galactose and galactitol concentrations. Toxic levels of Gal-1-P and galactitol may be responsible for the development of acute neonatal symptoms in patients with generalized and intermediate GALE deficiency [39]. These Gal-1-P levels range from > 30 mg/dL in patients with intermediate or peripheral deficiency to 170 mg/dL in patients with generalized deficiency [9].

### Recommendations and follow-up

**Fig. 3:**
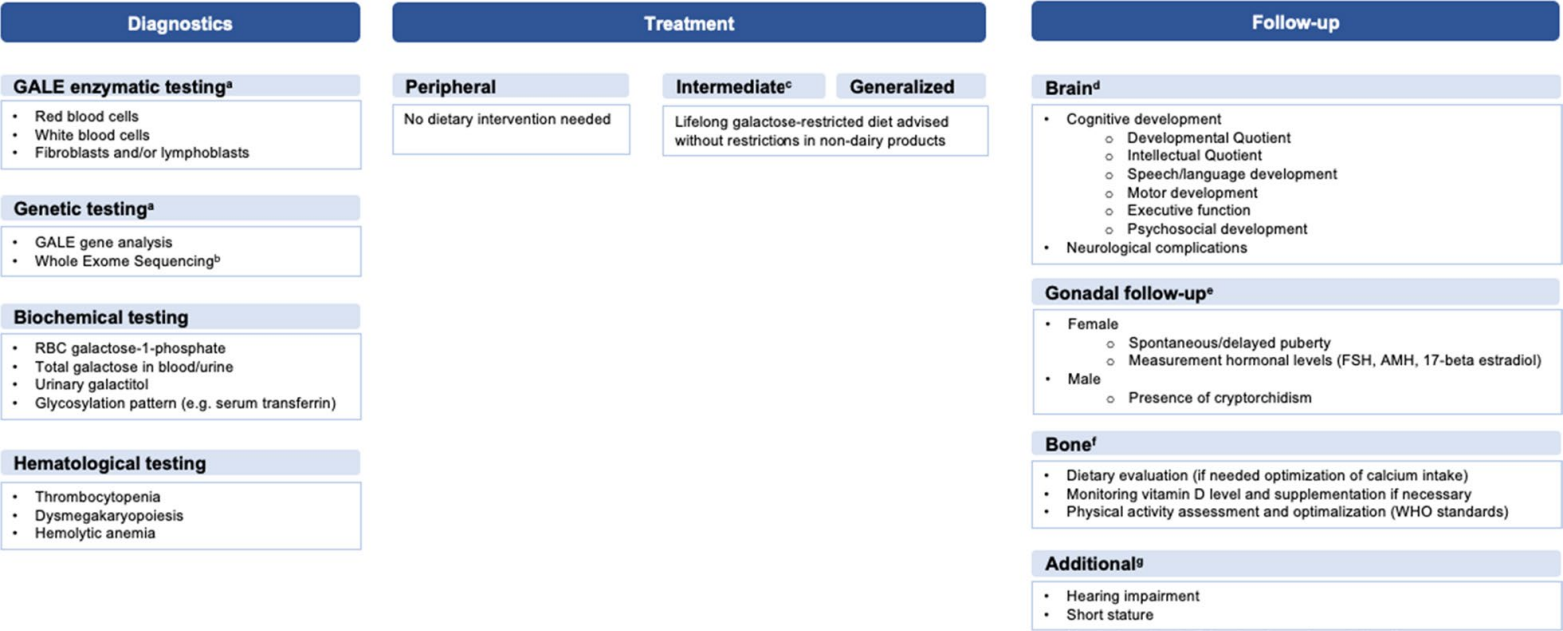
**Schematic recommendation for standardized diagnosis, treatment and follow-up in GALE deficiency.** These recommendations are based on the collected information from our study population and the international clinical guideline of classic galactosemia (CG). **a** GALE enzymatic and genetic testing is needed for classification (generalized, intermediate or peripheral). In the presence of genetic variants clearly associated with a peripheral/intermediate/generalized form, further enzymatic testing in non-peripheral cells is not needed. If the given genotype is uncertain, the whole work up of GALE enzymatic and genetic testing is advised. **b** It is recommended to perform a WES in consanguineous families or when other genetic conditions could be responsible for the genotype. **c** Evidence is lacking whether or not to start a galactose-restricted diet in patients with intermediate GALE deficiency. The long-term outcomes and effect of dietary intervention remain unclear. **d** Periodic brain follow-up is recommended. If learning disabilities, speech/language and/or motor and/or psychosocial problems are noted, adequate testing for in-depth assessment is advised. **e** Gonadal follow-up is recommended due to the gap of knowledge in this entity regarding possible gonadal disfunction. It is recommended to evaluate the presence of ovarian disfunction in females and the presence of cryptorchidism in males. **f** Bone health follow-up is advised to monitor periodically. Following the guidelines for CG, a DEXA-scan is recommended from the age of 8–10 years. **g** Hearing screening is recommended in the first year of life. Short stature has been regularly reported, so it is recommended to evaluate the length periodically. *Welling L, et al. Galactosemia Network (GalNet). International clinical guideline for the management of classical galactosemia: diagnosis, treatment, and follow-up. J Inherit Metab Dis. 2017;40(2):171–176.* https://doi.org/10.1007/s10545-016-9990-5.* Epub 2016 Nov 17. PMID: 27,858,262; PMCID: PMC5306419*

Standardized diagnosis, treatment and follow-up are recommended to truly clarify the phenotypic spectrum. When patients show multiple symptoms and GALE deficiency is suspected, exclusion of other genetic conditions related to these symptoms is helpful. Based on the current insights and gaps in knowledge of this rare entity, a schematic overview with recommendations for diagnosis, treatment and follow-up is created (Fig. 3).

### Study limitations

This study was limited by the small study population due to the low prevalence of the disease, the retrospective nature of data collection, and no standardized methods of follow-up.

## Conclusion

We described the phenotypic spectrum of 22 patients with GALE deficiency, 6 of whom were classified as generalized. In total 10 previously unpublished *GALE* variants were identified. Not only genetic variants and affected enzymatic tissues, but also the clinical picture, should be taken into account to classify the patient.

In many centers, additional enzymatic or genetic testing to better classify the deficiency and thus the follow-up is not part of common practice due to lack of facilities to measure GALE enzyme activities in other cells rather than RBC. It is important to distinguish among GALE patients who need dietary intervention (generalized and intermediate) versus those who probably do not (peripheral). In addition to the clinical picture, investigating abnormal glycosylation, such as serum transferrin, may be of help in the decision to start dietary galactose restriction or not. The systematic follow-up of the clinical and biochemical follow-up including long-term outcome of this group of patients should be standardized world-wide to gain a better understanding of this entity.

## Patients and methods

### Ethics statement

Rubio-Gozalbo et al. (2019) [23] described the establishment of the GalNet in 2012 and the implementation of an online patient registry (https://ecrf.ctcm.nl/macro/) including patients with galactosemia from several countries. The online patient registry was established in accordance with Good Clinical Practice and is following General Data Protection Regulation. The local ethics committee of the coordinating center (Maastricht University Medical Center + (MUMC +)) approved the study (application number METC 13–4-121.6/ab) and was subsequently approved by the participating partners. Patients’ data of centers not participating in the GalNet registry were collected with Collection Forms (see Additional file 3) with similar questions as in the online registry. All patients or their authorized representatives gave written patient consent for data collection and use for scientific publication.

### Patients

Data of 22 patients with GALE deficiency were collected between December 2014 and April 2022. Patients were classified as generalized or non-generalized GALE deficiency. Patients with known genotype and enzyme activities in different tissues were classified following the criteria formulated by Fridovich-Keil et al. (1993–2021) [9]. Patients who could not be categorized using these criteria, were classified based on their clinical picture and professional judgment of the treating physician. The category non-generalized included patients most likely to have peripheral or intermediate GALE deficiency (Fig. 1).

### Visualization of sites of new genotypic variants

Pymol (www.pymol.org) was used to design a cartoon representation of the crystal structure of human GALE in complex with UDP-glucose and NADH. PDB-entry 1EK6 was used [40] (Fig. 2). The splice site predictions were investigated using Alamut Visual Plus v.1.3 and Genomnis HSF Mutations Analysis Version 2.02.

### Statistical analysis

Data of GALE deficient patients were exported from the database in MACRO to SPSS. Descriptive analyses were used to calculate medians and ranges for continuous variables and percentages for categorical variables. Clinical outcomes were classified as absent or present.

## Supplementary Information

**Additional file 1**. Detailed overview of genotype and phenotype per patient.

**Additional file 2**. Comparison of generalized patients with the literature.

**Additional file 3**. Collection forms.

## Data Availability

The dataset supporting the conclusions of this article is included within the article and its additional files.

## Abbreviations

APTT: Activated partial thromboplastin time
CDG: Congenital disorder of glycosylation
DEXA: Dual-energy X-ray absorptiometry
EBV: Epstein Barr virus
GALE: UDP-galactose-4-epimerase deficiency
GalNet: International network of galactosemias
Gal-1-P: Galactose-1-phosphate
gnomAD: Genome aggregation database
NBS: Newborn screening
PHA: Phytohemagglutinin
POI: Primary ovarian insufficiency
PT: Prothrombin time
RBC: Red blood cell
UDP-gal: Uridine diphosphate galactose
UDP-glc: Uridine diphosphate glucose
UDP-GalNAc: Uridine diphosphate-*N*-acetyl-galactosamine
UDP-GlcNAc: Uridine diphosphate-*N*-acetyl-glucosamine
WES: Whole exome sequencing
WHO: World Health Organization
