# Supplementary material for: Galactose epimerase deficiency: lessons from the GalNet registry
Source: Orphanet J Rare Dis. 2022 Sep 2;17:331. doi: 10.1186/s13023-022-02494-4 (PMC9438182; doi:10.1186/s13023-022-02494-4)
Supplement: Supplementary file 3 — Additional file 3. Collection forms. [file 13023_2022_2494_MOESM3_ESM.pdf]

REGISTRY ENTRY	
**Important information**	
• Age of data collection	
• Informed consent yes/no	
**General information**	
• Gender	
• Ethnicity	
• Mutation(s)	
• Enzyme activity (%) and method used for measurement in red blood cells, fibroblasts, leukocytes and/or lymphoblasts.	
• Other mutations (*is a Whole Exome Sequencing performed?)*	
**Neonatal data**	
• Start diet date (age of onset)	
• Diagnosed with newborn screening	yes/no
• Acute neonatal illness	yes/no
• Encephalopathy<br>*(altered mental state: depressed consciousness with or without neurological signs)*	yes/no
• Cataract in newborn period	yes/no
• Bleeding diathesis<br>*(PTT/APTT abnormal)*	yes/no
• Infection in newborn period<br>*(Clinical signs of infection/sepsis)*<br>• If sepsis, blood culture data present?	yes/no
• Elevated liver enzymes<br>*(ALT, AST >30 U/L)*	yes/no
• Hypoglycemia<br>*(<2.6 mmol/L)*	yes/no
• Gal-1-P (peak)<br>*Define unity (umol/gr Hb, mg%, other)*	yes/no
**General follow up (most recent contact)**	
• Age of follow-up	
• Length *(cm)*<br>• Short stature (*outside TH range)*	
• Weight *(kg)*	
• Head circumference *(cm)*	
• Sensorinal deafness present?	
• Hematological diseases present<br>*(thrombocytopenia, leukopenia, anemia and platelet dysfunction)*	
**Metabolites**	
Urinary galactitol + unit *(recent, neonatal)*	
Total galactose in blood + unit *(recent, neonatal)*	
Transferrin IEF + unit *(before and after initiation diet)*	
**Brain follow up**	
• Brain follow-up yes/no	

• Imaging<br>*Specify imaging technique*	
• Developmental delay	yes/no → specify mental/motor/both and age of onset, still present?
• Language delay	yes/no<br>*Age of onset, still present?*
• Impairment in grammar	yes/no<br>*Age of onset, still present?*
• General motor abnormality	yes/no<br>*Age of onset, still present?*
• Impairment in vocabulary	yes/no<br>*Age of onset, still present?*
• Speech defect	yes/no<br>*Age of onset, still present?*
• Verbal dyspraxia	yes/no<br>*Age of onset, still present?*
• Dysarthria	yes/no<br>*Age of onset, still present?*
• Neuropsychological tests performed? Information on IQ-test available?	
• Other neurological symptoms<br>(ataxia, chorea, ballismus, temor, dystonia, athetosis, seizures, behavioral problems, ADHD, diagnose of autism spectrum disorder, depression, anxiety disorder, schizophrenia)	*Define which one(s) and age of onset, still present?*
**Gonads and reproduction – Female**	
• Gonads and reproduction follow-up yes/no	
• Delayed puberty<br>*(lack of breast development by age 13)*	Yes/no
• Spontaneous puberty	Yes/no<br>*Regular cycles? Age of onset?*
• Induced	Yes/no<br>*At what age?*
• Hormone replacement therapy	Yes/no<br>*At what age?*
• Primary ovarian insufficiency<br>*(<40 years, >40 months amenorrhea, 2 independent more than 1 month apart FSH levels in menopausal state)*	Yes/no
Recent measurements of FSH, estradiol, AMH?	
• Gonads imaging	Yes/no<br>*Which method? Result?*
• Tried to conceive	Yes/no<br>*Relationshipstatus?*
• Pregnancy yes/no	Yes/no<br>*Spontaneous pregnancy/assisted reproduction*
• Biological children	Yes/no
• Age mother at first delivery	*How many children in total*
**Bones**	
• Bone health follow-up yes/no, date and age	
• DEXA-scan (Lumbar spine Z-score or T-score)<br>*Date of measurement and age at that time*	

• Bone fractures	Yes/no
• Vitamine D test performed<br>*Vit D (25-hydroxy D3) measurement*<br>*Value in nmol/L*	Yes/no
• Vitamine D supplement<br>*Amount in mcrg/dayor IU*	
 Calcium	
• Target amount/day	
• Diet calcium supplements<br>*Amount in mg/day*	Yes/no
• Calcium intake	
• Physical activity<br>*Specify (according to WHO recommendations)*	Yes/no
 Diet	
• Data on diet yes/no	
• Infant formula used at diagnosis	
• Recommended diet completely lactose free	Yes/no
• Recommended diet restrict in:<br>- galactosides (peas, beans, legumes, cocoa, soya)<br>- any fruit and vegetables<br>- nucleoproteins (egg, liver, kidney)	Yes/no
• Does the recommended diet allow a specified amount of galactose in the diet<br>*Specify proximal amount in mg/dag*	yes/no
• Any other vitamines of supplements outside mentioned vitamine D and calcium<br>*Specify*	yes/no
• Is any type of chees derived from dairy milk allowed<br>*Specify type and name*	yes/no
• Increased galactose intake independently<br>*Specify*	yes/no
• Does the amount of daily allowed galactose increase with age<br>*- Purposely relaxed diet?*<br>*- At what age purposely relax diet*<br>*- How much extra galactose is aimed to give*<br>*- Reason for dietary relaxation*	yes/no
 Additional	
If you could not classify the patient as peripheral, intermediate or severe, what where the reasons why you did not perform any further analysis (f.e. enzyme activity or genetic analysis)	
